# Supplementary material for: Telomere dysfunction cooperates with epigenetic alterations to impair murine embryonic stem cell fate commitment
Source: eLife. 2020 Apr 16;9:e47333. doi: 10.7554/eLife.47333 (PMC7192583; doi:10.7554/eLife.47333)
Supplement: Transparent reporting form [file elife-47333-transrepform.docx]

***eLife’s* transparent reporting form**

We encourage authors to provide detailed information *within their submission* to facilitate the interpretation and replication of experiments. Authors can upload supporting documentation to indicate the use of appropriate reporting guidelines for health-related research (see [EQUATOR Network](http://www.equator-network.org/%20)), life science research (see the [BioSharing Information Resource](https://biosharing.org/)), or the [ARRIVE guidelines](http://www.plosbiology.org/article/info:doi/10.1371/journal.pbio.1000412) for reporting work involving animal research. Where applicable, authors should refer to any relevant reporting standards documents in this form.

If you have any questions, please consult our Journal Policies and/or contact us: [editorial@elifesciences.org](mailto:editorial@elifesciences.org).

**Sample-size estimation**

- You should state whether an appropriate sample size was computed when the study was being designed
- You should state the statistical method of sample size computation and any required assumptions
- If no explicit power analysis was used, you should describe how you decided what sample (replicate) size (number) to use

Please outline where this information can be found within the submission (e.g., sections or figure legends), or explain why this information doesn’t apply to your submission:

For the studies described, power analysis was not conducted and sample size was instead chosen based on 3 biological replicates for each experimental test group, and where possible multiple technical replicates within each biological replicate group. The details regarding the number and type of replicates for each experiment are indicated in the text, in the figure, or in the figure legend. Any exceptions are also explicitly indicated. For the ATAC-seq analysis, a minimum of two biological replicates were analyzed, with more detailed information included in the Materials and Methods and in the Source data files.

**Replicates**

- You should report how often each experiment was performed
- You should include a definition of biological versus technical replication
- The data obtained should be provided and sufficient information should be provided to indicate the number of independent biological and/or technical replicates
- If you encountered any outliers, you should describe how these were handled
- Criteria for exclusion/inclusion of data should be clearly stated
- High-throughput sequence data should be uploaded before submission, with a private link for reviewers provided (these are available from both GEO and ArrayExpress)

Please outline where this information can be found within the submission (e.g., sections or figure legends), or explain why this information doesn’t apply to your submission:

Biological replicates are defined in the Materials and Methods pertaining to each experimental series. Where appropriate, exclusion of samples is indicated either in the text, figure legend, or in the Source Data files provided for each Figure. The fastq and bedgraph files associated with the ATAC-seq data will be deposited with the Gene Expression Omnibus, and the GEO metadata form is included as a Source Data file.

**Statistical reporting**

- Statistical analysis methods should be described and justified
- Raw data should be presented in figures whenever informative to do so (typically when N per group is less than 10)
- For each experiment, you should identify the statistical tests used, exact values of N, definitions of center, methods of multiple test correction, and dispersion and precision measures (e.g., mean, median, SD, SEM, confidence intervals; and, for the major substantive results, a measure of effect size (e.g., Pearson's r, Cohen's d)
- Report exact p-values wherever possible alongside the summary statistics and 95% confidence intervals. These should be reported for all key questions and not only when the p-value is less than 0.05.

Please outline where this information can be found within the submission (e.g., sections or figure legends), or explain why this information doesn’t apply to your submission:

The method of statistical analysis performed for each experiment is indicated in the figure legends and in Materials and Methods under “Quantification and statistical analysis”, including type of error (SD), p-values, and other statistical tests. Additional raw data is included in Source Data files that accompany the figures.

(For large datasets, or papers with a very large number of statistical tests, you may upload a single table file with tests, Ns, etc., with reference to sections in the manuscript.)

**Group allocation**

- Indicate how samples were allocated into experimental groups (in the case of clinical studies, please specify allocation to treatment method); if randomization was used, please also state if restricted randomization was applied
- Indicate if masking was used during group allocation, data collection and/or data analysis

Please outline where this information can be found within the submission (e.g., sections or figure legends), or explain why this information doesn’t apply to your submission:

Two major groups define distinct genotypes of murine embryonic stem cells: wild-type (WT) and telomerase reverse transcriptase knockout cells (KO: *mTert*-/-). Within these two major groups, cells were subjected to a differentiation protocol that involved isolation of cells at different stages of differentiation via FACS, which are indicated with color codes (e.g. see schematic in Figure 1—Figure supplement 1). Finally, the two cell genotypes were further subjected to treatments with various compounds, and these groups are elaborated within the legends and text accompanying Figures 2-4. No masking was using during data collection or analysis.

**Additional data files (“source data”)**

- We encourage you to upload relevant additional data files, such as numerical data that are represented as a graph in a figure, or as a summary table
- Where provided, these should be in the most useful format, and they can be uploaded as “Source data” files linked to a main figure or table
- Include model definition files including the full list of parameters used
- Include code used for data analysis (e.g., R, MatLab)
- Avoid stating that data files are “available upon request”

Please indicate the figures or tables for which source data files have been provided:

Source data files are provided for:

- Figure 1 and related Supplemental figure 1

- Figure 2 and related Supplemental figure 1

- Figure 3 and related Supplemental figure 1

- Figure 4 and related Supplemental figure 1

The raw (fastq) and processed (.bdg) ATAC-seq data will be deposited via the Gene Expression Omnibus, and the DOI accession number will be provided. A summary table of these samples and the quality control performed on them is provided in two Source data files that accompany Figure 4.
